# Anakinra usage in febrile infection related epilepsy syndrome: an international cohort

**DOI:** 10.1002/acn3.51229

**Published:** 2020-12-04

**Authors:** Yi‐Chen Lai, Eyal Muscal, Elizabeth Wells, Nikita Shukla, Krista Eschbach, Ki Hyeong Lee, Marios Kaliakatsos, Nevedita Desai, Ronny Wickström, Maurizio Viri, Elena Freri, Tiziana Granata, Srishti Nangia, Robertino Dilena, Andreas Brunklaus, Mark S. Wainwright, Mark P. Gorman, Coral M. Stredny, Abdurhman Asiri, Khalid Hundallah, Asif Doja, Eric Payne, Elaine Wirrell, Sookyong Koh, Jessica L. Carpenter, James Riviello

**Affiliations:** ^1^ Baylor College of Medicine/Texas Children's Hospital Houston Texas USA; ^2^ Children's National Health System Washington District of Columbia USA; ^3^ Department of Pediatrics Section of Neurology Children's Hospital Colorado University of Colorado Aurora Colorado USA; ^4^ AdventHealth Child Neurology and Comprehensive Epilepsy Center Orlando Florida USA; ^5^ Neurosciences Department Great Ormond Street Hospital London UK; ^6^ Department of Women's and Children's Health Karolinska University Hospital Neuropediatric Unit Stockholm Sweden; ^7^ Childhood Neuropsychiatric Department University Hospital Maggiore della Carità Novara Italy; ^8^ Department of Pediatric Neuroscience FONDAZIONE IRCCS ISTITUTO NEUROLOGICO "CARLO BESTA" Milan Italy; ^9^ New York Presbyterian Hospital‐Weill Cornell Medical College NYC New York New York USA; ^10^ Clinical Neurophysiology Fondazione IRCCS Ca' Granda Ospedale Maggiore Policlinico Milan Italy; ^11^ Department of Neurosciences Rehabilitation, Ophthalmology, Genetics, Maternal and Child Health University of Genova Genova Italy; ^12^ Fraser of Allander Neurosciences Unit Royal Hospital for Children Glasgow UK; ^13^ Department of Neurology University of Washington Seattle Washington USA; ^14^ Department of Neurology Pediatric Multiple Sclerosis and Related Disorders Program Boston Children's Hospital Harvard Medical School Boston Massachusetts USA; ^15^ Prince Sultan Medical Military City (PSMMC) Riyadh Saudi Arabia; ^16^ Division of Neurology CHEO Research Institute Faculty of Medicine University of Ottawa Ottawa Canada; ^17^ Divisions of Neurology Department of Pediatrics Alberta Children’s Hospital Calgary Alberta Canada; ^18^ Divisions of Child and Adolescent Neurology and Epilepsy Department of Pediatric Neurology Mayo Clinic Rochester Minnesota USA; ^19^ Division of Neurology Department of Pediatrics Emory University School of Medicine Atlanta Georgia USA

## Abstract

Febrile‐infection related epilepsy syndrome (FIRES) is a devastating neurological condition characterized by a febrile illness preceding new onset refractory status epilepticus (NORSE). Increasing evidence suggests innate immune dysfunction as a potential pathological mechanism. We report an international retrospective cohort of 25 children treated with anakinra, a recombinant interleukin‐1 receptor antagonist, as an immunomodulator for FIRES. Anakinra was potentially safe with only one child discontinuing therapy due to infection. Earlier anakinra initiation was associated with shorter duration of mechanical ventilation, ICU and hospital length of stay. Our retrospective data lay the groundwork for prospective consensus‐driven cohort studies of anakinra in FIRES.

## Introduction

Febrile‐infection related epilepsy syndrome (FIRES) is a devastating neurological condition with significant mortality and morbidity.^1^, ^2^, ^3^ It represents a subset of children with new onset refractory status epilepticus (NORSE) in whom a febrile infection precedes the onset of seizures.^4^ Emerging evidence suggests that neuroinflammation may contribute to the pathologenesis.^5^, ^6^, ^7^ Despite improvements in pediatric intensive care unit (ICU) support and encouraging results from ketogenic diet for refractory status epilepticus, there are no immunomodulatory treatments for children with FIRES.

Experimental models of status epilepticus (SE) have implicated innate immunity as a potential etiology of seizure susceptibility.^8^, ^9^, ^10^ Microglial activation and monocyte infiltration have been observed in the brain following SE.^9^ Similarly, SE increases brain mRNA and protein levels of interleukin‐1 beta (IL‐1β).^8^, ^9^ Exogenous administration of IL‐1β enhances seizure susceptibility^8^, ^10^. Administration of an IL‐1 receptor antagonist (IL‐1ra) ameliorates the pro‐convulsant effects of IL‐1β,^10^ suggesting that IL‐1ra may represent a candidate therapy.

In support of IL‐1ra as a potential target, children with febrile SE have lower serum ratios of IL‐1ra levels to pro‐inflammatory cytokines (IL‐6, IL‐8).^11^ Hyperactive IL‐1β activity and functional IL‐1ra deficiency are observed in children with FIRES, which can be ameliorated with recombinant IL‐1ra (anakinra).^5^ Anakinra therapy appears to dampen seizures, facilitates the withdrawal of anesthetic agents, and is associated with favorable clinical outcome in two children with FIRES.^6^, ^7^ These cases raise the possibility that exogenous IL‐1ra may represent a promising FIRES therapy. Here we report the safety and potential efficacy of anakinra as an immunomodulator for FIRES in an international case series of 25 children.

## Methods

Children on anakinra were identified through the international McMaster Rheumatology List serve between October 2017 and February 2018; and by contacting individual institutions. Two subjects were published in previous case reports.^12^ Contributing institutions received approval from the respective Institutional Review Boards or Ethics boards. Site investigators determined FIRES diagnosis according to the proposed consensus definitions^4^ but excluded children with suspected autoimmune encephalitis. We collected demographics, clinical and laboratory parameters at the initial ICU admission, anakinra dosing and duration, adverse events, and functional outcomes using a standardized abstraction form (supplemental materials). The Pediatric Cerebral Performance Category (PCPC) was determined by the site investigators. Neuropsychological domain assessments were performed by the respective contributing centers and reported as normal or abnormal. In a subset of children (*n* = 15), local investigators determined electrographic and electroclinical seizure frequency immediately before and one week after anakinra treatment. Subsequently we dichotomized these children into those with greater than 50% seizure reduction (*n* = 11) and those without (*n* = 4). We evaluated the demographics, clinical characteristics, and outcomes using descriptive statistics. The results are reported as number (%) or median [interquartile range]. Pearson correlation was used to evaluate the association between the timing of anakinra initiation and duration of mechanical ventilation, ICU length of stay (LOS) and hospital LOS.

## Results

### Demographics and Diagnostic Work‐up

Most patients were male (68%) and the median age at FIRES diagnosis was 8 years [5.2–11 years] (Table 1). The majority of initial cerebrospinal fluid and brain imaging studies were normal. Elevations in cerebrospinal fluid and serum cytokine/neopterin levels were often found in those children who underwent comprehensive testing (Table 1).

**Table 1:** Patient demographics and clinical characteristics.

Subject	Age (yrs)	Gender	Ethnicity	Seizure semiology	MRI findings	CSF WBC (cells/mm^3^)	CSF RBC (cells/mm^3^)	CSF Protein (mg/dL)	CSF glucose (mg/dL)	⇑ CSF cytokines^1^	⇑ Serum cytokines^1^	⇑ CRP or ESR	⇑ ANA	⇑ thyroid antibody
1	5.6	Male	Middle Eastern	generalized & focal	‐inflammatory changes	‐	‐	‐	‐	IL‐6	‐	yes	no	No
2	9	Male	Caucasian	focal	‐changes c/w acute sz ‐inflammatory changes	1	‐	‐	‐	‐	‐	no	no	No
3	5	Male	Asian	generalized	‐changes c/w acute sz	‐	‐	‐	‐	‐	‐	yes	no	No
4	11	Male	Hispanic	focal	‐normal	3	0	38	64	IL‐1β, IL‐4, IL‐5, IL‐6, IL‐8, IL‐10, IFN‐γ		no	no	No
5	11	Female	Caucasian	focal	‐normal	1	1	27	59	‐	‐	yes	no	No
6	5	Male	Caucasian	focal	‐normal	3	655	35	83	‐	‐	yes	no	No
7	5	Female	African	multifocal	‐normal	0	0	12	94	‐	‐	no	no	No
8	7	Male	Caucasian	focal	‐normal	3	2	44	77	IL‐5, IL‐8, CXCL‐10	IL‐1β, IL‐6, IL‐8, CXCL‐9, CXCL‐10	yes	no	yes
9	12	Male	Other	generalized	‐normal	<5	<5	35	85	‐	‐	no	no	No
10	8	Male	Asian	Multi‐focal	‐normal	2	1	15	53	‐	‐	yes	no	No
11	11	Female	Caucasian	focal	‐inflammatory changes	6	1	33	69	‐	‐	yes	no	No
12	9	Female	Caucasian	focal	‐normal	0	0	25	52	none	‐	no	no	No
13	6	Female	Caucasian	generalized	‐inflammatory changes	0	0	68	97	none	‐	‐	no	No
14	9	Male	Middle Eastern	multifocal	‐changes c/w acute sz	0	4	19	54	none	‐	yes	no	No
15	6	Male	Hispanic	generalized	‐normal	0	2	28	74	‐	IL‐2	yes	yes	yes
16	15	Male	Hispanic	generalized	‐ischemic	3	310	28	72	none	TNF, IL‐6, neopterin	yes	yes	yes
17	8	Male	Other	multifocal	‐structural malformation	3	0	17	81	‐	‐	yes	no	No
18	8	Male	African	generalized	‐normal	10	1	49	66	‐	IL‐5, IL‐6, IL‐10	yes	yes	No
19	7	Male	Asian	generalized	‐normal	4	2	39	69	‐	IL‐6	no	no	No
20	5	Female	Hispanic	generalized	‐ischemic	2	2	44	96	‐	neopterin, IL‐6, IL‐10	no	no	No
21	14	Female	Asian	generalized	‐normal	2	0	36	70	‐	neopterin	no	yes	No
22	5	Female	Caucasian	generalized	‐normal	‐	4275	32	88	‐	‐	yes	no	No
23	16	Female	Asian	generalized	‐inflammatory changes	2	2	‐	‐	neopterin	IL‐2	yes	no	No
24	5	Male	Asian	generalized	‐normal	3	0	78	29	neopterin		no	no	No
25	4	Male	African	focal	‐changes c/w acute sz ‐inflammatory changes	1	1	‐	‐	neopterin	IL‐2, IL‐6	yes	no	No

AE, autoimmune encephalitis; ANA, antinuclear antibody; CRP, c‐reactive protein; CSF, cerebrospinal fluid; CXCL, chemokine (C‐X‐C motif) ligand; ESR, erythrocyte sedimentation rate; IFN, interferon; IL, interleukin; sz: seizures; TNF, tumor necrosis factor.

^1^ CSF and serum cytokine studies were not obtained in all subjects as indicated by the blank entries.

### Treatment Prior to Initiation of Anakinra

Prior to the initiation of anakinra, all children were treated with anesthetic agents to achieve seizure control (Table 2). Two children were on midazolam infusion alone; 5 on pentobarbital alone; and 18 on both infusions. All children received at least 4 additional anti‐seizure medications (ASMs), with 18 children (72%) having failed 7 or more agents prior to anakinra initiation. Nineteen children (76%) were on the ketogenic diet; 7 (28%) received cannabinoids. Corticosteroids and intravenous immunoglobulin (IVIG) were used in 22 children (88%). Of these 22 children, 11 (44%) received plasmapheresis and 5 (20%) rituximab.

**Table 2:** Treatment of super‐refractory status epilepticus and anakinra usage.

Subject	Duration of cEEG (days)	Continuous infusions	Burst‐suppression	Illness onset to pentobarbital (hours)	Pentobarbital duration (days)	# of ASMs	Adjunct therapies	Immune therapy	KD	Sz onset to anakinra (days)	Anakinra dose (mg/kg/day)	Concurrent immune therapies or KD	> 50% Sz reduction	Anakinra duration (days)	Adverse events
1	59	MDZ, pentob	yes	96	7	> 10	lidocaine, ketamine	steroid, IVIG	yes	17	10	steroid, KD	yes	19	Cytopenia
2	9	MDZ, pentob	yes	48	4	8	lidocaine, CBD, DBS	steroid, IVIG, plasmapheresis	yes	42	4	steroid, KD	no	> 114	None
3	‐	MDZ, pentob	yes	72	3	> 10	lidocaine, ketamine, CBD, DBS	steroid, IVIG, plasmapheresis	yes	21	10	steroid, KD	no	> 124	None
4	21	MDZ, pentob	yes	48	2	9	ketamine, hypothermia	steroid, IVIG, plasmapheresis	yes	14	12.2	KD	yes	83	Infection
5	16	MDZ, pentob	yes	36	8.5	9	ketamine	steroid, IVIG	yes	19	3	KD	yes	730	Infection
6	14	MDZ, pentob	yes	12	11	> 10	ketamine, hypothermia, VNS, isoflurane	steroid, IVIG, plasmapheresis	yes	18	4.7	KD	‐	2	None
7	10	MDZ, pentob	yes	24	4	> 10	lidocaine, ketamine	steroid, IVIG	yes	24	13	KD	‐	‐	None
8	60	MDZ, pentob	yes	96	7	8	ketamine	steroid, IVIG	yes	50	6.3	steroid, KD	‐	330	None
9	32	MDZ, pentob	yes	144	24	‐	propofol, ketamine, CBD	steroid, IVIG	yes	20	10	steroid, KD	‐	350	None
10	5	MDZ, pentob	yes	48	16	8	ketamine, hypothermia	steroid, IVIG	yes	23	2	KD	‐	7	Infection
11	43	MDZ, pentob	yes	96	17	> 10	propofol, ketamine	IVIG	yes	9	8	KD	yes	26	Infection
12	26	MDZ, pentob	yes	2	4	8	none	steroid, IVIG, rituximab	yes	15	3.3	KD	‐	14	None
13	9	MDZ, pentob	yes	8	2	7	none	none	no	5	10	no	‐	16	None
14	70	MDZ, pentob	yes	1	120	9	hypothermia	steroid, IVIG, rituximab	yes	20	5	KD	yes	120	Infection
15	27	pentob	yes	312	12	6	none	steroid, IVIG, plasmapheresis	no	32	7	no	yes	252	DRESS
16	36	MDZ, pentob	yes	48	25	6	none	steroid, IVIG, plasmapheresis	no	25	4	no	‐	420	infection, cytopenia, DRESS
17	24	MDZ, pentob	yes	14	9	> 10	hypothermia, CBD	steroid, IVIG	yes	6	3.8	KD	no	9	None
18	19	MDZ	no	‐	‐	4	none	steroid, IVIG	no	20	3.2	no	yes	90	None
19	27	pentob	yes	190	12	5	none	steroid, IVIG, plasmapheresis	no	12	7	steroid	yes	270	Infection
20	141	pentob	yes	24	106	> 10	propofol, ketamine, CBD	steroid, IVIG, plasmapheresis, rituximab	yes	34	9	KD	yes	200	infection, ⇑ LFT
21	44	pentob	yes	336	17	4	none	steroid, IVIG	yes	12	7.5	KD	yes	183	Infection
22	‐	pentob	yes	30	16	6	clonidine	None	yes	1	4.7	KD	‐	14	None
23	‐	MDZ	no	‐	‐	9	ketamine, CBD	steroid, IVIG, plasmapheresis, rituximab	yes	30	2	KD	yes	97	Infection
24	8	MDZ, pentob	yes	44	12	> 10	ketamine	steroid, IVIG, plasmapheresis	no	14	5	steroid	‐	2	DRESS
25	74	MDZ, pentob	yes	60	17	> 10	ketamine, CBD	steroid, IVIG, plasmapheresis, rituximab	yes	33	5	KD	no	5	None

ASM, anti‐seizure medication; CBD, cannabinoids; cEEG, continuous electroencephalography; DBS, deep brain stimulation; DRESS, drug reaction with eosinophilia and systemic symptoms syndrome; IVIG, intravenous immunoglobulin; KD, ketogenic diet; LFT, liver function test; MDZ, midazolam; pentob, pentobarbital; Sz, seizure.

### Anakinra therapy

Anakinra was started at a median of 20 days [14–25 days] after the onset of seizures. Initial median anakinra dose was 3.8 mg/kg per day [3–5 mg/kg per day] and a final median dose 5 mg/kg per day [4–9 mg/kg per day] (Tables 2, 3). The median duration of anakinra therapy was 86 days [13–257 days] with 12 children (48%) continuing the treatment following hospital discharge. Nine children (36%) had infections prior to anakinra initiation whereas 10 (40%) had infections following treatment (Table 2). Three children (12%) developed drug reaction with eosinophilia and systemic symptoms syndrome (DRESS), which was treated with the addition or escalation of corticosteroids. All recovered without complications. Two children (8%) developed cytopenias that eventually resolved without specific intervention. Anakinra was discontinued in only one child due to infection.

**Table 3:** Clinical outcomes.

	All subjects (n = 25) median [IQR] or N (%)	> 50% seizure reduction at 1 week (n = 11) median [IQR] or N (%)	No seizure reduction (n = 4) median [IQR] or N (%)
Seizure onset to anakinra initiation (days)	20 [14 ‐ 25]	19 [12 ‐ 30]	27 [13.5 ‐ 37.5]
Final anakinra dose (mg/kg/d)	5 [4 −9]	7 [3.2 ‐ 9]	4.5 [3.9 ‐ 7.5]
Ketogenic diet use	19 (76)	8 (72)	4 (100)
Number of ASMs	9 [7 ‐ >10]	9 [5 ‐ >10]	> 10 [9 ‐ >10]
Mechanical ventilation (days)	36 [21 ‐ 54]	35.5 [22 ‐ 44]	50.5 [35.5 ‐ 111.5]
ICU length of stay (days)	54 [25 ‐ 69]	47.5 [34 ‐ 108]	66 [43.5 ‐ 70]
Hospital length of stay (days)	73.5 [35 ‐ 118]	108 [60 ‐ 131]	93 [48.5 ‐ 119]
Number of infections before Anakinra	0 [0 ‐ 1]	1 [0 ‐ 1]	0 [0 ‐ 2]
Number of infections after Anakinra	0 [0 ‐ 2]	2.5 [0 ‐ 5]	0
Respiratory	9 (36)	7 (63.6)	0
Urinary tract infection	6 (24)	6 (54.6)	0
Others	4 (16)	3 (12)	0
Number of ASMs at discharge^1^	3 [3 ‐ 4]	3 [2 ‐ 5]	3 [2 ‐ 4]
PCPC at discharge^2^			
Normal	2 (8)	0 (0)	0
Mild disability	1 (4)	0 (0)	0
Moderate disability	7 (28)	5 (45.5)	1 (25)
Severe disability	4 (16)	2 (18.2)	1 (25)
Persistent vegetative state/coma	3 (12)	2 (18.2)	1 (25)
Dead	3 (12)	0 (0)	1 (25)
PCPC at follow up^1^			
Normal	2 (9.1)	0	0
Mild disability	4 (18.2)	2 (18.2)	0
Moderate disability	6 (27.3)	4 (36.4)	2 (66.7)
Severe disability	4 (18.2)	3 (27.3)	0
Persistent vegetative state/coma	1 (4.5)	0	1 (33.3)
Dead	0	0	0
Neuropsychological domain assessment^1^			
Motor deficit	11 (50)	5 (45.5)	3 (100)
Attention deficit	17 (77.3)	9 (81.8)	3 (100)
Memory deficit	12 (54.5)	7 (63.6)	3 (100)
Executive function deficit	15 (68.2)	8 (72.7)	3 (100)
Speech deficit	13 (59.1)	7 (63.6)	3 (100)
Return to school^1^	12 (54.5)	6 (54.5)	2 (66.7)
regular class	2 (16.7)	0	0
with accommodations	5 (41.7)	3 (50)	0
special education	5 (41.7)	3 (50)	2 (100)

ASM, anti‐seizure medication; IQR, interquartile range; PCPC, pediatric cerebral performance category

^1^ All subjects: *n* = 22;> 50% seizure reduction: *n* = 11; no seizure reduction: *n* = 3

^2^ All subjects: *n* = 22;> 50% seizure reduction: *n* = 11; no seizure reduction: *n* = 4

### Outcomes

The median time on mechanical ventilation was 36 days [21–54 days], ICU LOS was 54 days [25–69 days], and hospital LOS was 73.5 days [35–118 days]. Median number of ASMs at discharge was 3 [3–4]. Earlier anakinra initiation after seizure onset was associated with shorter duration of mechanical ventilation, and ICU and hospital LOS (*r* = 0.46 (*P* = 0.03), *r* = 0.50 (*P* = 0.01) and *r* = 0.48 (*P* = 0.03), respectively). Amongst children with available seizure frequency data (*n* = 15), 11 exhibited> 50% seizure reduction at 1 week of anakinra treatment. Although there were no statistical differences due to the small sample size, several observations were notable between children with and without seizure reduction (Table 3). The median interval between seizure onset and anakinra initiation was 19 days [12–30 days] in children with seizure reduction; 27 days [13.5–37.5 days] in children without seizure reduction. The median duration of mechanical ventilation and ICU LOS were 35.5 days [22–44 days] and 47.5 days [34–108 days] in children with seizure reduction; 50.5 days [35.5–111.5 days] and 66 days [43.5–70 days] in children without seizure reduction.

Three children died (12%), all from withdrawal of support due to the persistent super‐refractory SE and the expected poor neurological outcome. The timing of withdrawal varied from 1 to 18 days following anakinra initiation. The median length of follow‐up for the surviving children with available information (*n* = 17) was 321 days [219–420 days]. Six had no or minimal disability (PCPC 1‐2), 6 had moderate disability (PCPC 3), and 5 had severe disability or vegetative state (PCPC 4‐5). All surviving children had drug resistant epilepsy. The most prominent neuropsychological deficits at follow‐up were in attention (*n* = 17), executive functioning (*n* = 15), and speech (*n* = 13). Twelve children returned to school, 10 required academic accommodations or special education classes.

## Discussion

Here we describe the safety and potential efficacy of anakinra therapy in the largest international retrospective cohort of FIRES patients. Infections, transaminitis, and neutropenia represent potential anakinra‐associated side effects. In our cohort, the prevalence of infection before and after anakinra initiation were comparable; and only one had transaminitis. While increased eosinophil count is a known side effect of anakinra, DRESS syndrome has not been associated with anakinra in rheumatic conditions.^13^ In FIRES patients, DRESS may likely reflect an underlying immune dysregulation, as evidenced by some children with FIRES meeting criteria for hemophagocytic lymphohistiocytosis characterized by pathologic immune activation.^14^ None of the patients experienced adverse outcomes due to DRESS syndrome or cytopenia with conservative management. However, these complications may present more serious challenges with increasing anakinra utilization for the treatment of FIRES.

Early anakinra initiation was associated with shorter mechanical ventilation days, ICU and hospital LOS, and possibly seizure reduction, suggesting that innate immunity may contribute to the pathology of FIRES. Specifically, elevated IL1‐β levels have been described in the cerebrospinal fluid and the serum of three children with FIRES; and the functional IL1‐ra deficiency in the cerebrospinal fluid of one children.^5^ Exogenous IL‐1ra administration ameliorated the functional IL1‐ra deficiency *ex vivo*, providing a rationale for using recombinant IL‐1ra (anakinra) as an immunomodulatory treatment for FIRES.^5^ We found that administering anakinra to children with FIRES may indeed be beneficial, which supports hyperactive IL1‐β activity and/or functional IL‐1ra deficiency as significant pathological factors underlying FIRES.

There are several limitations to our study that are inherent in a retrospective case series and highlight current knowledge gaps in the field. Although early anakinra initiation may be beneficial as demonstrated in this study, we were unable to ascertain the optimal therapeutic window for anakinra treatment. The duration of therapy was highly variable with 10 children receiving < 1 month of treatment. In contrast, response to anakinra in the rheumatologic conditions such as systemic juvenile arthritis has been assessed no earlier than 4–12 weeks from initiation of therapy.^15^ These limitations, coupled with patient and other clinical factors, likely contributed to the lack of improved long‐term neurological outcome in this study. Nevertheless, our findings provide additional support for anakinra as a potential immunomodulator for patients with FIRES. Prospective studies are necessary to understand: a) the optimal timing, dosing and duration of anakinra therapy, b) rational biologic correlates of anakinra response, and c) safety and efficacy of anakinra and ASMs.

## Author contributions

All authors were engaged in either formulation of data abstraction tool or compiling of patient de‐identified data. All authors critically appraised all versions of the manuscript. Drs. Muscal, Lai, and Riviello conducted data analysis. Cases from MK have previously been published individually.

## Conflict of interest

Dr. Wainwright is a member of the clinical advisory board for Sage Therapeutics. Dr. Brunklaus has received speaker honoraria from Biocodex, Zogenix, Nutricia and Encoded Therapeutics. Dr. Wirrell has received honoraria from Biocodex, other from Biomarin, other from Mallinckrodt. Dr. Koh reported funding from Sobi external from this manuscript. Dr. Riviello is a consultant for Biomarin and the CLN2 North American Advisory Board.
